# Tuning and validation of a virtual mechanical testing pipeline for condylar stress fracture risk assessment in Thoroughbred racehorses

**DOI:** 10.1098/rsos.241935

**Published:** 2025-05-07

**Authors:** Soroush Irandoust, Chris Whitton, Corinne Henak, Peter Muir

**Affiliations:** ^1^ Surgical Sciences, School of Veterinary Medicine, University of Wisconsin-Madison, Madison, WI 53706, USA; ^2^ Mechanical Engineering, College of Engineering, University of Wisconsin-Madison, Madison, WI 53706, USA; ^3^ Equine Centre, Melbourne Veterinary School, University of Melbourne, Werribee, Victoria 3030, Australia; ^4^ Biomedical Engineering, College of Engineering, University of Wisconsin-Madison, Madison, WI 53706, USA; ^5^ Orthopedics and Rehabilitation, School of Medicine and Public Health, University of Wisconsin-Madison, Madison, WI 53705, USA

**Keywords:** Thoroughbred racehorses, third metacarpal bone, condylar stress fracture, functional adaptation, finite element analysis

## Abstract

Condylar stress fracture of the third metacarpal/metatarsal bone (MC3/MT3) in Thoroughbred racehorses is a common catastrophic injury, putting both the racehorse and the jockey at risk. Microdamage forms in the distal MC3 and may result in strain elevation and higher risk of stress fracture, directly and through focal osteolysis resulting from the associated damage repair by remodelling in the condylar parasagittal grooves (PSG). Standing computed tomography (sCT) is a practical screening tool for detecting fatigue-induced structural changes, but clinical interpretation remains subjective. The goal of this study was to develop and validate an sCT-based, subject-specific finite element analysis pipeline for virtual mechanical testing of the distal MC3. Twelve (*n* = 12) MC3 condyles with available *ex vivo* strain were selected. Half of the specimens were used for tuning Young’s modulus in fatigue sites, and the other half were used to evaluate the validity of the pipeline in predicting PSG strain. The tuned model improved the prediction of strain and predicted higher strain in bones with PSG lysis over the untuned model, which is an essential feature for a diagnostic screening tool. The presented pipeline can assist clinicians with the interpretation of sCT-detectable structural changes and, ultimately, with the assessment of condylar fracture risk in racing Thoroughbreds.

## 1. Introduction

Condylar stress fracture of the metacarpophalangeal/metatarsophalangeal (MC3/MT3, fetlock) joint is a common catastrophic injury in Thoroughbred racehorses worldwide and a major cause of euthanasia [1–3]. It is the second most common serious musculoskeletal injury in the United States, following proximal sesamoid bone fracture [4,5]. In addition, jockey falls associated with catastrophic horse injuries are more likely to result in serious and life-changing disability to the rider than jockey falls due to other reasons. Jockeys were ~160–200 times more likely to fall and get injured when they rode a horse that had a catastrophic musculoskeletal injury [6,7]. Focal microdamage, incomplete fractures, and preexisting resorption at sites prone to condylar fracture have been observed in the fractured and contralateral nonfractured limbs of Thoroughbred racehorses [8–15], confirming that condylar fractures of the MC3/MT3 are fatigue-induced stress fractures [11]. Thoroughbreds with a completely displaced condylar stress fracture have a decreased prognosis for racing after surgery [16–19]; hence, prevention of these injuries and associated horse death and jockey injury from falls is an important focus.

Large cyclic loads and microdamage accumulation in the distal end of the MC3/MT3 trigger new trabecular bone modelling and subchondral sclerosis [20,21], which are thought to help unload fatigued bone, allowing focal remodelling to repair damage. Site-specific microdamage in bone can be resorbed through bone remodelling, but because repetitive high loads suppress bone resorption, damage accumulation often outpaces this process [22]. Such an imbalance can result in subchondral bone fatigue injury (SBI) in the parasagittal grooves (PSG), visible as focal osteolysis lesions on standing computed tomography (sCT) imaging. Both focal microdamage and osteolysis have been shown to compromise the mechanical properties of distal metacarpal subchondral bone *ex vivo* [23,24].

sCT is a practical, noninvasive racehorse screening method with high sensitivity for detecting structural changes associated with fatigue damage [25–27]. Despite the high sensitivity of sCT for the detection of these changes, clinical interpretation is still challenging, and an objective classifier for condylar fracture risk assessment is lacking. Thus, combining sCT with physics-based predictive models has the potential to improve the identification of MC3/MT3 bones at risk of fracture.

CT-based, subject-specific finite element (FE) modelling is a helpful tool for virtual, noninvasive mechanical testing and has been extensively used for biomechanical assessment of human hips and spines [28–30]. In the fetlock of Thoroughbred racehorses, although prior micro-CT-based FE analysis (FEA) has shed light on the biomechanical properties of the subchondral bone [31], there is currently an urgent need for an sCT-based FEA pipeline to assist clinicians with condylar fracture risk assessment. Therefore, the goal of this study was to develop and validate an sCT-based, subject-specific FEA pipeline for virtual mechanical testing of the distal MC3 in Thoroughbred racehorses.

## 2. Material and methods

### 2.1. Specimen selection

A library of 12 MC3 condyles (*n* = 12) from 11 thoracic limb specimens, with available surface strain data under quasi-static loading of these condyles from previous *ex vivo* mechanical testing [23], was used for this study. Specimens were collected from Thoroughbred racehorses that were euthanatized because of catastrophic racetrack injuries, including contralateral MC3 fracture through the PSG. Age, sex, and training history of the specimens were not available. These condyles included specimens with (*n* = 4, PSG-SBI group) and without (*n* = 8, CTRL group) sCT-detectable focal lysis in the PSG of the distal end of the MC3 bone. The existence of fatigue damage in the PSG of the specimens in the PSG-SBI group was previously confirmed following soft tissue dissection [23,25].

### 2.2. Standing computed tomography acquisition and calibration

The sCT scans of the frozen limbs were acquired using an Equina^®^ scanner (Asto CT Inc., Middleton, WI, USA) at exposure of 160 kVp and 8 mA, with a slice thickness of 0.55 mm. An electron density phantom (model 062M; CIRS Inc., Arlington, VA, USA) with four calcium hydroxyapatite (HA) plugs with densities of 200, 800, 1250, and 1750 mg cm^−3^, was scanned using the same scanner at the same exposure settings to find the equivalent radiological or CT density, $\rho_{CT}$ ($mg_{HA}\;cm^{-3}$; electronic supplementary material, figure S1):


(2.1) $$\begin{array}{c} \rho_{CT}=\left(CT\;number-93.0710\right)/1.0123. \end{array}$$

### 2.3. Segmentation of the distal MC3

**Figure 1.**
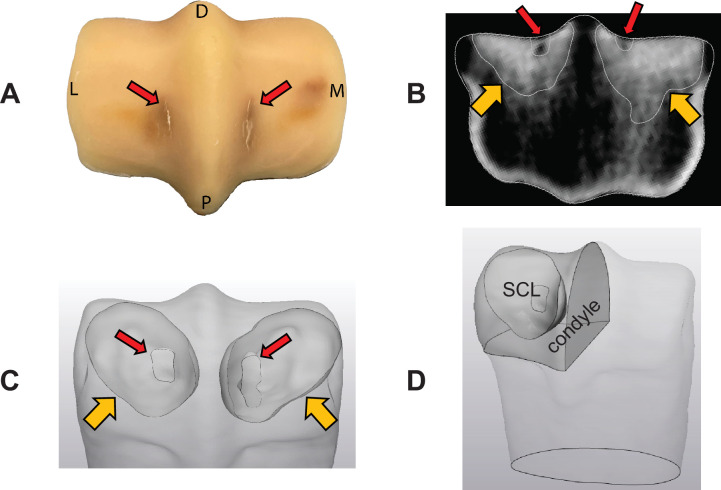
Segmentation of the sclerotic and lytic regions. Adaptive and damage factors were used for tuning Young’s modulus in sclerotic and lytic regions, indicated with orange (wide) and red (thin) arrows, respectively. Fatigue cracks are visible on the palmar aspect of the parasagittal grooves of the condyle PSG-SBI-1 after dissection and removal of soft tissue (A). Sclerotic and lytic regions of PSG-SBI-1 were segmented from the standing CT image (B,C). A representation of the measurement of the volume of a condyle with its sclerotic region is shown (D).

Mimics (v.26) was used to segment the distal MC3 (figure 1). Using a gradient-based segmentation tool, the external surface of the bone was detected semi-automatically for all individual sCT image slices sequentially. After creating the three-dimensional distal MC3, using morphology operations, one pixel was eroded from the external surface to make sure it was tight on the cortex before smoothing and importing it into 3-matic (v.18) for further processing. An anatomical coordinate system was established by first finding the long axis of the bone using a cylinder fit. The transverse plane was then constructed perpendicular to the long axis of the bone. The sagittal plane was established perpendicular to the transverse plane through the dorsal and palmar aspects of the sagittal ridge. The frontal plane perpendicular to the transverse and frontal planes was then constructed. The distal 2.5 inches (63.5 mm) of the MC3 was isolated, and a homogeneous triangular mesh with an edge length of 0.25 mm was generated.

### 2.4. Density–modulus relationship

The mechanical properties of bone have a strong correlation with its density [32], and this relationship can be used to predict heterogeneous bone mechanical properties. First, CT density from equation (2.1) was converted to ash density using the data from Knowles *et al*. [33]:


(2.2) $$\begin{array}{c} \rho_{ash}=0.8772\times \rho_{CT}+0.07895. \end{array}$$

In equation (2.2), $\rho_{ash}$ and $\rho_{CT}$ are expressed in $g\;cm^{-3}$. The Young’s modulus was then calculated using the experimental data collected from horse limbs by Moshage *et al*. [34]:


(2.3) $$\begin{array}{c} E=3378\times \rho_{ash}^{1.52}. \end{array}$$

In equation (2.3), E is expressed in MPa and $\rho_{ash}$ in $g\;cm^{-3}$.

### 2.5. Modulus tuning for the subchondral sclerotic and lytic regions

**Figure 2.**
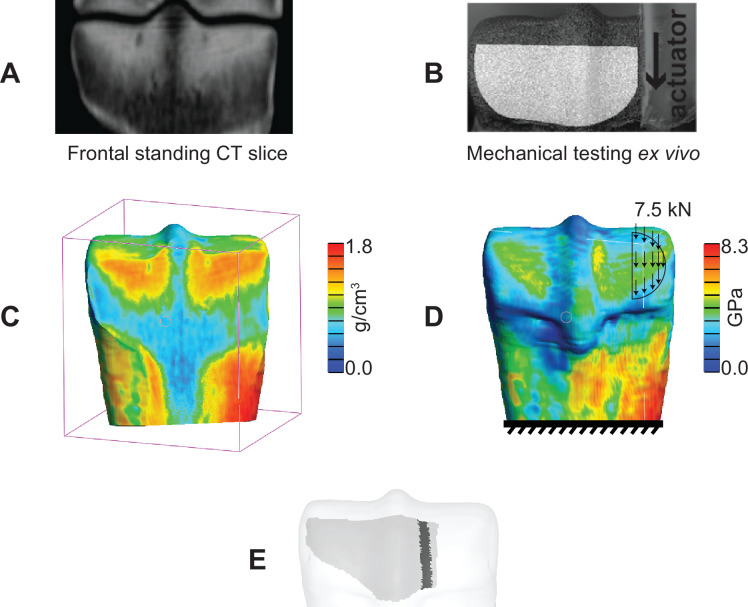
Finite element (FE) modelling pipeline. (A) Standing CT (sCT) imaging was used for segmentation of the distal MC3 volume. (B) Surface strain data from previous *ex vivo* testing data were used as the reference data for comparison with the FE results. (C) Element-wise heterogeneous HA density was captured from the sCT image set. (D) Young’s modulus was assigned to individual elements, and the *ex vivo* loading and boundary conditions were replicated in the model. (E) The surface for which *ex vivo* mechanical testing data were available (light grey) and the associated PSG region (dark grey) were registered on the three-dimensional segmented bone end for comparison of the surface strain.


Using the available *ex vivo* mechanical testing data (electronic supplementary material, figure S2), the mean of the maximum principal strain in the PSG (a 3 mm wide strip centred at the groove that was used to compare strains *ex vivo*; figure 2E) at peak displacement was compared with the FE-predicted strain in the same region. The mean was selected to avoid the error that can result from absolute maximum values. In our preliminary investigations, we found that FE predictions of PSG strain using the density–modulus relationship in equation (2.3) did not match well with the experimental results. This was likely due to the complex mechanical properties of the fetlock subchondral bone in actively training and racing Thoroughbreds. Microdamage is not captured with clinical CT due to resolution limitations, and although the density–modulus relationship for the subchondral equine MC3 bone has been reported before at the microscale [31], there are no available data at the macroscale. To address these two main limitations, sclerotic and lytic regions, if present, were segmented separately (figure 1). To separate these regions from the rest of the distal MC3, a threshold of HU=1500, equivalent to 1390 $mg_{HA}\;cm^{-3}$, was used based on the location of the secondary peaks in the voxel density histograms of the distal MC3 that indicate the adaptive response, as previously discussed [23]. Trabecular regions with HU>1500 were defined as the sclerotic region (SCL), and isolated focal regions in the PSG with HU<1500, if present, were defined as the lytic region (LYS). A modified density–modulus relationship was used by incorporating an adaptive factor for the sclerotic region ($-1.00<A_{SCL}<0.50$) and a damage factor for the lytic region ($0.50<D_{LYS}$), as shown in equations (2.4) and (2.5):

(2.4) $$\begin{array}{c} E_{SCL}=\left(1-A_{SCL}\right)\times 3378\times \rho_{ash}^{1.52}, \end{array}$$

(2.5) $$\begin{array}{c} E_{LYS}=\left(1-D_{LYS}\right)\times 3378\times \rho_{ash}^{1.52}. \end{array}$$

To find the optimal $A_{SCL}$, the normalized volume of the sclerotic region was measured ($V_{SCL}=V_{sclerosis}/V_{condyle}$) and half of the CTRL condyles were chosen (*n* = 4) in such a way to include condyles with small and large sclerotic volumes and low and high *ex vivo* PSG maximum principal strain. For each of the four models, all $A_{SCL}$ values between −1.00 and 0.50 were used at 0.05 intervals to find the optimal value that resulted in an average PSG maximum principal strain closest to the experimentally measured values. Since it has been shown that bones with larger sclerotic volumes contain higher degrees of microdamage in the subchondral bone [21], the optimal $A_{SCL}$ was tuned as a function of $V_{SCL}$ (figure 1).

To find the optimal $D_{LYS}$, half of the condyles with PSG-SBI (*n* = 2) were selected, and all $D_{LYS}$ values between 0.50 and 0.95 were used at 0.05 intervals to find the optimal value that resulted in an average PSG maximum principal strain closest to the experimentally measured values. Due to the limited number of condyles in this group (*n* = 2), the average of the optimal $D_{LYS}$ for these two limbs was determined as the optimal value. The calibrated $A_{SCL}$ was used for defining the Young’s modulus in the sclerotic regions in these limbs.

### 2.6. Discretization, loading, and boundary conditions

FE models were created in FEBio Studio (v.2.3) with 0.25 mm edge length linear tetrahedral elements in the osteolytic and sclerotic regions and 1 mm edge length linear tetrahedral elements elsewhere. Adequate mesh density was confirmed via mesh convergence analysis (electronic supplementary material, figure S3). A Poisson’s ratio of 0.3 and sCT-based Young’s modulus were assigned to individual elements using the density–modulus relationships detailed in §§2.4 and 2.5.

The proximal end of the model was fully constrained, and a 7.5 kN load, uniformly distributed as nodal forces, was applied to the palmar surface of the medial condyle at 60° and 30° with respect to the frontal and transverse planes, respectively, replicating the experimental loading conditions [23] (figure 2).

### 2.7. Model validation

The optimal $A_{SCL}$ and $D_{LYS}$ were used to model the other half of the CTRL and PSG-SBI condyles to validate the modelling approach. Predicted average PSG maximum principal strains were compared with the experimental results as the gold standard to determine the accuracy of the predicted PSG strain by the tuned virtual mechanical testing pipeline. Predictions of the tuned model were compared with those of the non-tuned model, where *A*_SCL_ and *D*_LYS_ are zero.

## 3. Results

### 3.1. Tuned adaptive factor for the sclerotic regions

**Figure 3.**
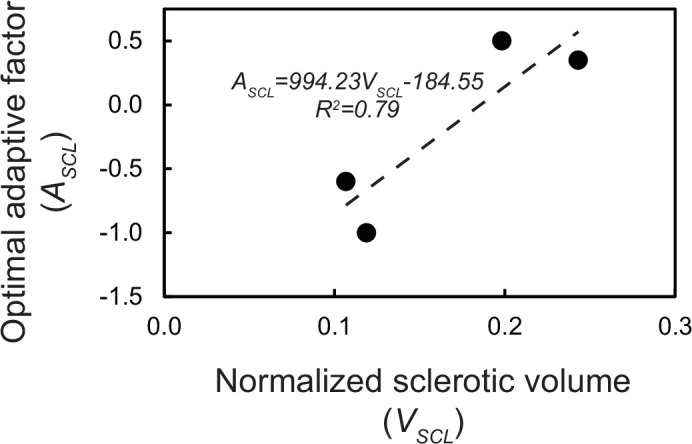
Adaptive factor *A*_SCL_ showed a positive relationship with the normalized subchondral sclerotic volume *V*_SCL_. This relationship was found by linear regression.

The group of four CTRL condyles that included low and high *V*_SCL_ (CTRL-2 and CTRL-3) and *ex vivo* PSG strain (CTRL-1 and CTRL-5) were identified (electronic supplementary material, table S1). The optimal *A*_SCL_ was a unique value for each of these condyles, ranging from −1.00 to 0.50. Models of condyles with smaller *V*_SCL_ required a negative *A*_SCL_ for their strain predictions to match the *ex vivo* results, and condyles with larger *V*_SCL_ required a positive *A*_SCL_ to capture the higher degree of microdamage in the subchondral sclerotic bone, which results in a compromised Young’s modulus. Additionally, condyles with smaller *V*_SCL_ were less sensitive to changes in *A*_SCL_ (electronic supplementary material, table S2). To find the optimal *A*_SCL_ function, it was plotted against *V*_SCL_, which showed a positive correlation (figure 3), and a linear regression was made, resulting in the following relationship:


(3.1) $$\begin{array}{c} A_{SCL}=994.23\times V_{SCL}-184.55. \end{array}$$

Equation (3.1) was used for the adaptive factor to adjust the Young’s modulus (equation (2.4)) in the sclerotic regions in all the following models. Using all eight CTRL specimens and their optimal *A*_SCL_ values had a small influence on this relationship (electronic supplementary material, figure S4).

### 3.2. Tuned damage factor for the lytic regions

To select two of the PSG-SBI condyles for finding the optimal *D*_LYS_, PSG-SBI-3 was excluded due to its complex joint disease pathology, which included palmar osteochondral disease (POD) as well as PSG-SBI. PSG-SBI-2 was excluded due to a very small overlap of the fatigue damage in the PSG area with the bone surface from which strain was measured [23]. Condyles PSG-SBI-1 and PSG-SBI-4 were selected, and the optimal *D*_LYS_ for these condyles was found to be 0.80 and 0.50 respectively (electronic supplementary material, tables S3 and S4). As a result, an average constant value of 0.65 was determined as the overall optimal *D*_LYS_.

### 3.3. Model validation

**Figure 4.**
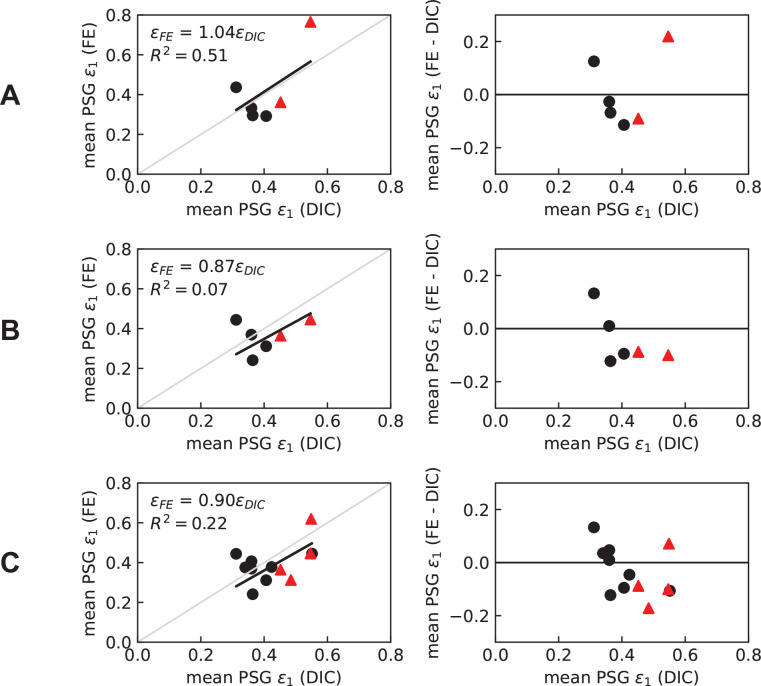
Comparison of the FE-predicted and the *ex vivo*-measured (DIC) mean PSG strain. (A) Tuned model on the validation group. (B) Non-tuned model on the validation group. (C) Non-tuned model on all specimens. Triangular (red) and circular (black) datapoints represent the PSG-SBI and CTRL condyles, respectively. The non-tuned pipeline leans towards underestimating PSG strain (slope < 1), especially in bones with PSG-SBI, and explains very little variance in the experimental data (small *R^2^*). These limitations were resolved by the tuned modelling pipeline, which predicts PSG strain with about 4% error on average.

The other half of the condyles were modelled to investigate the validity of the PSG strain predictions (electronic supplementary material, table S5; figure 4). The optimal *A*_SCL_ and *D*_LYS_ determined in §§3.1 and 3.2 were used to assign the Young’s modulus to the sclerotic regions and the lytic regions, if present.

Tuning of Young’s modulus improved predictions of PSG strain over the non-tuned model (figure 4). A linear regression between the predicted PSG strain and the measured *ex vivo* strain yielded a slope of 1.04 and *R^2^* = 0.51 (figure 4A). The difference between FE-predicted and *ex vivo* PSG strain was smaller than 0.12 except for PSG-SBI-3 (electronic supplementary material, table S5; figure 4A), which also had POD.

**Figure 5.**
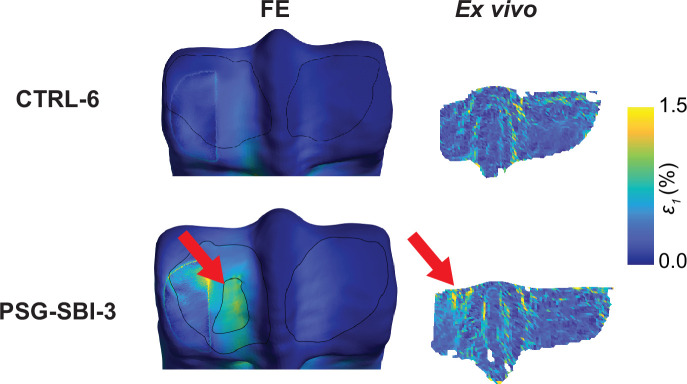
*Ex vivo*-measured and FE-predicted surface strain for the condyles CTRL-6 and PSG-SBI-3. *Ex vivo* strain data were not available for the dorsal aspect of the condyles and the area under the indenter. Red arrows show the location of the strain concentration in the PSG in the vicinity of the lysis that matches between the *ex vivo* and the FE-predicted results.

Surface strain fields were compared between *ex vivo* and FEA and showed good qualitative agreement (figure 5; electronic supplementary material, figure S5). PSG-SBI-3 showed maximum principal strain values exceeding 1% in some regions of the PSG closer to the lysis. In this condyle, strain concentration was observed on the joint surface over the dorsal edge of the lytic volume. There was no sign of high strain concentration in the PSG of the CTRL condyles used for the model validation investigation. Overall, condyles with PSG-SBI showed higher PSG strain by qualitative comparison (electronic supplementary material, figure S5).

## 4. Discussion

The virtual mechanical testing pipeline presented here is the first model, to our knowledge, that can predict subject-specific load-induced strain on the joint surface of the distal MC3 bone in Thoroughbred racehorses using sCT-based FEA, validated against *ex vivo* mechanical testing data. While sCT imaging has revolutionized racehorse screening by making it practical for routine injury prevention diagnostic imaging, the quantified objective prediction of the biomechanical behaviour of the subchondral bone provides valuable additional information that has the potential to assist clinicians in interpreting fatigue-induced subchondral structural changes in the fetlock and prevent potential underestimation of condylar stress fracture risk [25]. With the growing use of sCT, longitudinal screening of horses is now feasible, enabling assessments of lesion progression or healing of PSG-SBI.

Tuning the Young’s modulus in the subchondral sclerotic and severely damaged bone resulted in three major improvements compared to the non-tuned model, which assigns the same density–modulus relationship for the whole distal MC3. It improved the prediction of PSG strain, explained a larger variation in the PSG strain measured *ex vivo*, and decreased the bias towards under-predicting PSG strain in bones with PSG-SBI. It is critical for this virtual mechanical testing pipeline to properly predict the high PSG strain in bones with PSG-SBI that were measured *ex vivo* [23], to prevent false-negative diagnosis of Thoroughbreds that are at a potentially higher risk of condylar stress fracture, when the goal of subject screening is to minimize the risk of serious injury to both the Thoroughbred horses and their jockeys.

The subchondral sclerotic volume was used as a biomarker for the extent of fatigue damage in the sclerotic bone in our approach, since microdamage is not captured within the resolution of clinical CT. Model tuning indicated that the subchondral sclerotic bone volume is positively associated with fatigue-induced mechanical compromise in this region. This observation is aligned with previous findings, which showed a positive association between bone volume fraction in the PSG and the degree of microdamage in this region [35].

Accurate prediction of bone strain is contingent on using a suitable density–modulus relationship, especially in the subchondral sclerotic bone. There are a limited number of studies that provide the density–modulus relationship for the equine MC3 at the macroscale [34,36]. However, neither provides data on the subchondral sclerotic bone. Establishing a specific density–modulus relationship for this region has the potential to improve the strain prediction accuracy. Despite the lack of such a site-specific relationship, the Young’s modulus modified by defining adaptive and damage factors in the present study could partially fill this gap in knowledge and successfully increased the accuracy of bone strain prediction.

While the presented study provides a platform for biomechanical assessment within the quasi-static elastic deformation, future investigations on yield strength, post-yield behaviour, and ultimate strength of MC3 equine bones, especially in the subchondral sclerotic region, could provide more insight into their plastic behaviour and toughness [37]. The subchondral sclerotic MC3 bone, with bone volume fractions like cortical bone, loses its energy dissipation capabilities moving away from the joint surface in the oblique dorso-palmar direction, despite having higher stiffness [38]. Although cortical bone has significantly lower toughness than trabecular bone [39], the difference in energy dissipation capabilities between sclerotic and non-sclerotic equine subchondral MC3 bone is a current gap in knowledge. Such information could improve the prediction of stress fracture initiation and may enable the prediction of its propagation path [40]. In addition to enabling the prediction of the load to failure on a patient-specific basis, another possible alternative next step is to compare the volume of subchondral bone that contains strain/stress levels above a critical threshold, which has been shown to be a strong predictor of the fatigue life [41].

The main limitation of this study is the small number of samples with *ex vivo* mechanical testing data available for tuning and validation, mainly due to the burden of such experiments [23]. This forced tuning of the damage factor in the lytic regions to a constant value, while the association between the optimal damage and potentially some anatomical features, such as the lytic volume, could have been investigated, as was done when tuning the adaptive factor for the sclerotic bone. Another limitation is not including a bone with isolated POD in this study. Bones with POD have more extensive subchondral sclerosis [23,42–44], which results in a larger normalized sclerotic volume. Condyle PSG-SBI-4 had both POD and PSG-SBI pathologies with a high sclerotic volume. This resulted in the highest allowed adaptive factor (the highest allowed reduction in the Young’s modulus) for the sclerotic region, which could have caused an overestimation of PSG strain and the highest error compared to *ex vivo* strain data. Although our *ex vivo* data revealed the highest PSG strain in this particular case, coexistence of multiple fatigue pathologies in the fetlock joint is possible, and care should be taken when interpreting the predicted surface strain by the presented modelling pipeline. One limitation pertaining to the methodology presented here is the definition of homogeneous adaptive and damage factors in the sclerotic and lytic regions, while there are higher degrees of microdamage closer to the articular surface; however, such data are not available due to the resolution of clinical sCT. Future studies focused on spatial quantification of microdamage within these regions and its association with other fatigue biomarkers, such as the normalized sclerotic volume, can help alleviate this challenge.

## 5. Conclusion

In conclusion, the validated virtual mechanical testing pipeline presented here predicts subchondral MC3 PSG strain *ex vivo* on a subject-specific basis. Based on prior literature linking elevated strains to bone fracture, this has the potential to develop into an objective mechanical assessment tool to identify racing Thoroughbreds at high risk of condylar stress fracture. Our modelling pipeline can be applied to populations of racing Thoroughbreds to compare predicted strain with the associated clinical risk assessment. Accordingly, a classifier such as a logistic regression model can be built for the classification of racehorses to low- and high-risk categories for condylar stress fractures. The classifier can then be clinically validated in the scenario where a racehorse develops a catastrophic condylar stress fracture or lameness associated with fetlock SBI. In future work, our modelling pipeline could also be used in longitudinal clinical studies. Longitudinal screening of racehorses could inform understanding of how the PSG lesion and subchondral sclerotic volume change over time, addressing a critical gap in clinical knowledge. This type of screening approach has the potential to reduce the incidence of serious or fatal injury in horses and produce an associated reduction in serious injuries to racehorse jockeys. This translational knowledge is also expected to advance stress fracture injury prevention in human athletes.

## Data Availability

Experimental data are available in [23] and at Dryad [45].Standing computed tomography images are available at Dryad [46]. Supplementary material is available online [47].
